# Fairness versus efficiency: how procedural fairness concerns affect coordination

**DOI:** 10.1007/s10683-017-9540-5

**Published:** 2017-09-04

**Authors:** Verena Kurz, Andreas Orland, Kinga Posadzy

**Affiliations:** 1 School of Business, Economics and Law, Department of Economics, University of Gothenburg, 405 30 Gothenburg, Sweden; 2 Department of Economics, University of Potsdam, August-Bebel-Str. 89, 14482 Potsdam, Germany; 3 Division of Economics, Department of Management and Engineering, Linköping University, 581 83 Linköping, Sweden

**Keywords:** Coordination, Correlated equilibrium, Recommendations, Procedural fairness, Volunteer’s Dilemma, Experiment, C72, C91, D63, D83

## Abstract

We investigate in a laboratory experiment whether procedural fairness concerns affect how well individuals are able to solve a coordination problem in a two-player Volunteer’s Dilemma. Subjects receive external action recommendations, either to volunteer or to abstain from it, in order to facilitate coordination and improve efficiency. We manipulate the fairness of the recommendation procedure by varying the probabilities of receiving the disadvantageous recommendation to volunteer between players. We find evidence that while recommendations improve overall efficiency regardless of their implications for expected payoffs, there are behavioural asymmetries depending on the recommendation: advantageous recommendations are followed less frequently than disadvantageous ones and beliefs about others’ actions are more pessimistic in the treatment with recommendations inducing unequal expected payoffs.

**Electronic supplementary material:**

The online version of this article (doi:10.1007/s10683-017-9540-5) contains supplementary material, which is available to authorized users.

## Introduction

Coordination problems are frequent in everyday interactions. Consider a situation at work in which exactly one volunteer is needed for serving on a workplace committee or writing the report from a meeting. If one person volunteers, everyone will benefit from the report being written or from a well-functioning committee. However, volunteering is time-consuming and hence costly to the individual, so everyone prefers someone else doing it. In order to avoid a situation where no one volunteers (or too many sign up), the employees have to solve a coordination problem. Where no formal rules are established, such problems can be solved with the help of some mechanism—for example, by a social norm determining who should do the task (the youngest team member or the oldest, etc.), via a coin toss, or by a third party (e.g. the boss) picking the one who should do the job. However, such a mechanism might lead to some individuals contributing more often, while others frequently escaping from investing time. External mechanisms that imply different likelihoods of being picked as a volunteer across individuals might be perceived as unfair by both the picked volunteer and the beneficiaries.

In this paper, we examine experimentally whether procedural fairness plays a role for how well individuals are able to solve a coordination problem in a two-player Volunteer’s Dilemma (Diekmann 1985). In this game, it is sufficient that one member of a group volunteers in order to provide a public good and make everyone better off. However, volunteering induces costs that are specific to the provider. As it does not matter who volunteers, two pure-strategy efficient Nash equilibria but no dominant strategies exist.^1^ Without any additional mechanism, coordination on one of the equilibria can be difficult to achieve. Both under-provision (no one volunteers) and over-provision (too many people volunteer) constitute inefficient outcomes resulting from coordination failure.^2^ To overcome the coordination problem, we give participants action recommendations—either to play the costly action or to abstain from it. Both players know their own recommendation and also which recommendation the other player receives. By allowing individuals to condition their action on the recommendation they receive, coordination on an efficient outcome can be achieved even without direct communication. Correlated equilibria become attainable, which can raise expected payoffs above Nash equilibrium payoffs (Aumann 1974, 1987). While fairness certainly plays a role in many experimental games, a coordination game like the Volunteer’s Dilemma is especially suitable to study how fairness of an external mechanism affects behaviour, as there are no dominant strategies and large potential efficiency gains.

We manipulate the fairness of the recommendation procedure by varying the probabilities with which subjects receive a recommendation to volunteer.^3^ By doing so, we alter the expected payoffs of following recommendations between subjects. Our definition of procedural fairness focuses on players’ sensitivity towards those differences in expected payoffs. We evaluate the behaviour of advantaged and disadvantaged individuals with respect to following recommendations, the resulting coordination rates, and earnings both in comparison to situations without any recommendations and compared to a fair mechanism.

Previous experimental studies show that fair action recommendations often enhance efficiency. Van Huyck et al. (1992) find that subjects follow public, non-binding announcements if they do not conflict with payoff-dominance. Furthermore, subjects are more likely to follow announcements if they induce equal average payoffs compared to unequal average payoffs across a session. Croson and Marks (2001) study a threshold public good game and find that individual recommendations about each subject’s contribution increase efficiency in contrast to a situation without recommendations. Duffy and Fisher (2005) show that potentially irrelevant public announcements about market conditions can help subjects coordinate on “sunspot equilibria” in laboratory financial markets. Cason and Sharma (2007) show that private action recommendations are followed if players believe that their counterparts will follow as well. Duffy and Feltovich (2010) find that subjects follow private recommendations if they are payoff-enhancing compared to the Nash equilibrium, but do not follow recommendations resulting in payoffs lower than in Nash equilibrium.

Most previous experiments use mechanisms that treat players symmetrically, such that all players can expect the same payoffs before the recommendation is realized. We will examine if a coordination mechanism that systematically puts one party at a disadvantage implies efficiency losses compared to such fair mechanisms. Our work is closely related to Anbarci et al. (2017), who investigate the impact of payoff-asymmetry on following recommendations in Battle of the Sexes games. By varying payoff asymmetry and the availability of recommendations between treatments, they study whether recommendations that point at both Nash equilibria with equal probability improve coordination. They find, as predicted, that subjects are less likely to follow recommendations in games with higher payoff asymmetry. While Anbarci et al. vary the payoff matrix of the underlying game and keep the probabilities of the recommendations of the two equilibria equal, we keep the payoffs constant and use the probabilities with which we recommend each of the two Nash equilibria to manipulate procedural fairness across treatments. By doing so, expected payoffs of following a recommendation before it is realized vary between players.

Previous experimental work suggests that people do not only care about ex-post inequality of outcomes, but also about procedural fairness, of which ex-ante inequality in expected payoffs is an important aspect (Bolton et al. 2005; Krawczyk and Le Lec 2010; Brock et al. 2013; Linde and Sonnemans 2015). Closely related to our experiment is Bolton et al. (2005), who study ultimatum games where first moves are decided by lotteries. Via the calibration of the lottery, the expected value of the proposal is manipulated. They find that low proposals are more acceptable if the lottery is judged fair compared to a lottery that is biased towards the disadvantageous outcome. Theoretical models such as by Trautmann (2009), Krawczyk (2011) or Saito (2013) account for the empirically observed importance of procedural fairness by incorporating expected payoffs into the utility function.

We investigate whether inequality in expected payoffs affects the efficiency of action recommendations as a coordination mechanism with the help of three experimental treatments: subjects play a Volunteer’s Dilemma and receive either (1) no recommendations, (2) efficient recommendations that induce *equal* expected payoffs as long as both subjects follow the recommendations, and (3) efficient recommendations that induce *unequal* expected payoffs as long as both subjects follow.^4^ This allows us to answer the following questions: Does inequality in expected payoffs matter for the decisions to follow action recommendations? Do differences in expected payoffs reduce efficiency gains of external recommendations in a coordination game? And does the behaviour of advantaged and disadvantaged individuals differ with regard to following the recommendations?

Our results show that most of the subjects are more concerned about efficiency and potential gains from coordination rather than about differences in expected payoffs. Recommendations increased efficiency in comparison to a treatment without any coordination mechanism in both the case with equal and unequal expected payoffs. We find that subjects are more likely to follow recommendations that give secure payoffs, even if they are disadvantageous, i.e. induce the equilibrium with a comparatively lower payoff for the player. While there were no significant differences in following recommendations between treatments, we find differences in individuals’ beliefs about others’ actions between treatments.

## Analytical framework

### Action recommendations in the Volunteer’s Dilemma

Table 1 presents the basic set-up of the two-player Volunteer’s Dilemma. A public good is provided if at least one player volunteers. Both players decide simultaneously between *X* (volunteer) and *Y* (not volunteer).^5^ Each player receives *a* if at least one of them volunteers and 0 if no one volunteers. A volunteer bears the cost *c*, $c>0$. Both players are better off when volunteering compared to a situation in which no one volunteers: $a>a-c>0$.

**Table 1:** Payoff matrix of the Volunteer’s Dilemma

	Player 2	
	$X\ \text {(volunteer)}$	$Y\ \text {(not volunteer)}$
Player 1		
*X* (volunteer)	$a-c$, $a-c$	$a-c$, *a*
*Y* (not volunteer)	*a*, $a-c$	0, 0

The game has no dominant strategy. There are two pure strategy Pareto-efficient Nash equilibria (NE), (*X*, *Y*) and (*Y*, *X*), in each of which one of the players volunteers and the other does not, granting the payoff $a-c$ to the volunteer who plays *X* and *a* to the player playing *Y*. However, this equilibrium requires Nash conjectures, i.e., players having correct beliefs about other players’ actions.

Furthermore, the game has a mixed strategy Nash equilibrium (MNE), in which each of the players volunteers with probability $1-\frac{c}{a}$ and takes no action (*Y*) with probability $\frac{c}{a}$. The expected payoff for each player in the MNE is

1 $$\begin{aligned} \pi _{Nash}^{e}=a-c. \end{aligned}$$

The introduction of direct, private action recommendations can improve coordination by helping to avoid over- and under-provision. Given that both players know which recommendation the other one receives, they can correlate their strategies via the recommendations given: either player 1 receives a recommendation to play *X* and player 2 to play *Y*, or the other way round. If both players follow these recommendations, inefficient outcomes (*X*, *X*) and (*Y*, *Y*) are avoided and one of the efficient outcomes (*X*, *Y*) or (*Y*, *X*) is achieved. Correlated equilibria (CE) that raise expected payoffs above Nash payoffs become attainable. If the distribution of recommendations to both players is common knowledge, each player can calculate expected payoffs of a recommendation mechanism for herself and the other player (Aumann 1974, 1987).

### Procedural fairness

We use the distribution of recommendations to vary the expected payoffs between players. Let the probability of player 1 receiving recommendation *Y* and player 2 receiving recommendation *X* be denoted with *p*, $p>0$. Given our set of possible recommendations, the probability that player 1 will get recommendation *X* and player 2 will get recommendation *Y* equals $1-p$. Under the assumption that both players believe that the other one will follow the recommendation, no one has an incentive to deviate after the recommendation is realized, since a unilateral deviation would decrease her payoff. Hence, any convex combination of equilibria suggestions (*X*, *Y*) and (*Y*, *X*) constitutes a CE, independent of the value of *p*.

Assuming the other player will follow her recommendation, expected payoffs from following for player 1 are:

2 $$\begin{aligned} \pi _{1}^{e}=pa+(1-p)(a-c)=a-(1-p)c, \end{aligned}$$

and for player 2:

3 $$\begin{aligned} \pi _{2}^{e}=p(a-c)+(1-p)(a)=a-pc. \end{aligned}$$

Equations 2 and 3 show that correlating their strategies via following the action recommendations is individually rational for both players, as expected payoffs from a strategy to follow the recommendation are higher than the expected payoff from playing the MNE. If both players follow recommendations, the sum of expected payoffs is raised above the sum of NE payoffs.

Expected payoffs from a CE vary with the probability the two action recommendations are given. As can be seen from Eqs. 2 and 3, expected payoffs depend on the value of *p*. If $p=0.5$, both players can expect

4 $$\begin{aligned} \pi _{1,2}^{e}=a-0.5c \end{aligned}$$

as equilibrium payoffs.

For any value of *p* different from 0.5, expected payoffs from following recommendations will differ between player 1 and player 2. Differences in expected payoffs have been identified as an important aspect of procedural fairness. In contrast to outcome fairness models, such as models developed by Fehr and Schmidt (1999), Charness and Rabin (2002) or Bolton and Ockenfels (2000), where the difference in payoffs to be received matters for decision-making, individuals who care about procedural fairness take additional factors into account, such as expected payoffs or the feasibility of an equal split. For example, Trautmann (2009) develops a procedural fairness model based on the Fehr–Schmidt model, but replaces differences in realized payoffs by differences in *expected* payoffs in the utility function. Besides absolute payoffs received, individuals care both about advantageous and disadvantageous inequalities in expected payoffs, but disutility from disadvantageous inequality is higher.

We adopt this definition of procedural fairness as differences in expected payoffs. As we keep the game’s underlying payoff structure constant across treatments, individuals who are purely motivated by distributional fairness should not base their decision to follow a recommendation on the value of *p*. In contrast, if players care about procedural fairness, *p* as a determinant of expected payoffs becomes relevant for decision-making. For $p>0.5$, player 1’s expected earnings will be greater than player 2’s, as the likelihood of receiving a recommendation “*Y*” (not to volunteer) is higher than receiving recommendation “*X*” (to volunteer). If a player cares about procedural fairness, and disutility from inequality in expected payoffs outweighs utility gains from the increase in expected payoffs, he will not follow recommendations. As aversion towards disadvantageous procedures is usually assumed to be higher than aversion towards advantageous procedures, it can be expected that disadvantaged players follow the recommendations less frequently.

## Experimental design

Table 2 shows the normal form of the Volunteer’s Dilemma game that subjects play. The payoff structure with $a=10$ and $c=5$ captures situations with high gains to both parties if one volunteers, high costs for the volunteer and zero payoffs to both parties when no one volunteers, and is in line with previous experimental work on the Volunteer’s Dilemma (Rapoport 1988; Diekmann 1993).

**Table 2:** The experimental calibration of the Volunteer’s Dilemma

	Player 2	
	*X*	*Y*
Player 1		
*X*	5, 5	5, 10
*Y*	10, 5	0, 0

In each session, 24 subjects participate. One half of the subjects is randomly assigned to the role of player 1; the rest of the subjects take the role of player 2. The role does not change during the experiment. The game is repeated for 30 rounds without any feedback between the rounds. In each round, subjects in the role of player 1 are randomly matched into pairs with subjects in the role of player 2. This matching procedure keeps the number of independent observations high and prevents subjects from developing strategies depending on past behaviour (e.g. subjects in Duffy et al. 2017 alternate when being repeatedly matched with the same partner).

The experiment has a between-subject design and consists of three treatments. In our first treatment, to which we will refer as *Baseline*, subjects play a standard Volunteer’s Dilemma game without any action recommendations. It serves as a benchmark to evaluate the effectiveness of the coordination mechanisms in other treatments.

Action recommendations are introduced in the remaining two treatments. In treatment *CD50*, equal probabilities are assigned to the two pure-strategy NE, which leads to the same number of recommendations to volunteer for both players. This treatment’s primary purpose is to measure the changes in coordination in comparison to the *Baseline* treatment. In the third treatment (*CD90*), different probabilities are assigned to action recommendations leading to the two pure-strategy NE. The desired NE for player 1, (*Y*, *X*), is recommended with probability 0.9 and the NE that puts player 2 at an advantage (*X*, *Y*) is recommended with probability 0.1. Thus, player 2 receives three advantageous recommendations (*Y*), while player 1 receives 27 such recommendations. This treatment allows us to study the effects of inequality in expected payoffs on coordination rates and efficiency. Table 3 summarizes our treatments and the expected payoffs to both players in each treatment. Expected payoffs are 5 points if the MNE is played. When action recommendations are followed, they increase to 7.5 points for both players in *CD50*, and to 9.5 and 5.5 points for player 1 and 2 respectively in *CD90* (7.5 points on average).

**Table 3:** Summary of the experimental design

Treatment	Recommendation	Expected payoff player 1	Expected payoff player 2
*Baseline*	None	5	5
*CD50*	$\hbox {P}(X,Y)=0.5$,	7.5	7.5
	$$\hbox {P}(Y,X)=0.5$$		
*CD90*	$\hbox {P}(X,Y)=0.1$,	9.5	5.5
	$$\hbox {P}(Y,X)=0.9$$		

Each round has the same structure. In all treatments, we present players with the normal form of the game on-screen. In the treatments with a coordination mechanism, *CD50* and *CD90*, subjects are also shown the probabilities of receiving each recommendation, their own recommendation for the round, and the recommendation their counterpart receives. The series of recommendations subjects receive were randomly generated before the experiments and are the same across sessions of a treatment. The series of recommendations for player 1 in both treatments can be found in the electronic supplementary material. Subjects do not receive any feedback about outcomes or past behaviour of other players until the very end of the experiment.

The experiment has a neutral framing. On-screen and in the printed instructions, subjects in the other role are called “the participant you are matched with”. Player 1 is called “Red participant”, player 2 “Blue participant”. The possible actions of the players are called *X* and *Y*. The coordination mechanism is called “recommendation” and we explain its working and consequences extensively in the instructions.^6^ It is displayed on the screen with the sentence “the recommendation is: ...”, directly above the field where subjects enter their decision.

After the experiment, we elicited risk preferences with an investment task proposed by Gneezy and Potters (1997). Subjects were endowed with 10 points (each point worth 10 euro cents) and had to decide about an investment in a risky asset. The asset had a probability of 0.5 of being successful: in this case it paid 2.5-fold the invested amount. With a probability of 0.5, the asset was not successful and the invested amount was lost.^7^ Subjects could invest any integer between 0 and 10 into the asset.

Furthermore, socio-demographic information was collected in a questionnaire after the experiment (age, gender, field of study, number of semesters in university). We also conducted two tests to account for possible effects of personality on behaviour, the Big Five personality traits (the BFI-S by Gerlitz and Schupp 2005) and Locus of Control (the IEC itinerary by Rotter 1966 in a German translation by Rost-Schaude et al. 1978). In the *CD50* and *CD90* treatments, two questions about the recommendations were included. Firstly, we elicited the beliefs about following behaviour of the participants in the other role (“Do you think that the participants in the other role followed the recommendation?”). The answer could be given on a scale with four items: all participants followed the recommendation, most participants followed it, most did not follow the recommendation, nobody followed the recommendation.^8^ Answers were summarized into a binary variable taking the value 1 if subjects answered that they believed other player always or most of the time followed the recommendation, and 0 otherwise. Subjects were also asked whether or not they felt disadvantaged by the recommendations.

Only after filling in the questionnaire, subjects were presented with the actions chosen by themselves and by the participant they were matched with in each round, the two randomly chosen rounds for the payment, and the payoffs from the risk elicitation task. The rounds chosen for payoff were the same for all subjects within a session. The exchange rate was 0.75 euros per point. Average total payoffs were 13.87 euros (including a show-up fee of 4 euros), with a minimum of 4.50 euros and a maximum of 21.50 euros. Payoffs were rounded up to the next full ten cents.

We conducted three sessions of each treatment, and in total 216 subjects participated. The experiments were conducted in MELESSA, the Munich Experimental Laboratory for Economic and Social Sciences, in January 2015. Each session lasted between 60 and 75 min. Instructions were read out loud and were available on paper throughout the experiment. To make sure that subjects understood the instructions, a computer-based quiz was conducted and the experiment only started after all subjects answered all control questions correctly. Subjects had the opportunity to individually ask questions (which rarely happened). All subjects answered the quiz correctly. We did neither exclude subjects from the experiment nor observations from the analyses. Full instructions for the *CD50* treatment with a screen-shot and control questions of all treatments can be found in the electronic supplementary material. The experiment was programmed in z-Tree (Fischbacher 2007) and participants were recruited via the ORSEE recruitment software (Greiner 2004).

## Hypotheses

We hypothesize that the existence of a coordination mechanism increases coordination and hence the earnings of players, as found in previous studies (for example, Cason and Sharma 2007; Duffy and Feltovich 2010). However, it is unclear how procedural fairness concerns affect the efficiency of action recommendations as a coordination mechanism. If preferences for payoff maximization are stronger than procedural fairness concerns, we will observe higher coordination rates than without recommendations, even when the coordination mechanism is unfair. On the other hand, if procedural fairness concerns are stronger than efficiency concerns, individuals will disregard the coordination mechanism. This lets us formulate the following hypotheses:

### **Hypothesis 1**

Coordination rates and earnings in treatments with recommendations that induce *equal* expected payoffs (*CD50*) are higher than in treatments without recommendations (*Baseline*).

### **Hypothesis 2a**

Coordination rates and earnings in treatments with recommendations that induce *unequal* expected payoffs (*CD90*) are higher than in treatments without recommendations (*Baseline*), if payoff maximization concerns are stronger than procedural fairness concerns.

Alternatively:

### **Hypothesis 2b**

Coordination rates and earnings in treatments with recommendations that induce *unequal* expected payoffs (*CD90*) are not higher than in treatments without recommendations (*Baseline*), if procedural fairness concerns are stronger than payoff maximization concerns.

Findings from experimental studies on procedural fairness show that individuals are less likely to accept biased procedures, even if this is connected with forgoing monetary payments (see for example Bolton et al. 2005). Hence, we predict that people are less likely to follow recommendations if they induce inequality in expected payoffs, in contrast to the case when they induce equal expected payoffs.

### **Hypothesis 3**

Coordination rates and earnings in treatments with recommendations that induce *equal* expected payoffs (*CD50*) are higher than in treatments with recommendations that induce *unequal* expected payoffs (*CD90*).

The frequency of coordination on one of the pure strategy NE in the treatments with coordination mechanism stems from individuals’ propensity to follow recommendations. We predict that individuals are less likely to accept recommendation procedures (i.e. follow recommendations) that systematically favour one of the players.

### **Hypothesis 4a**

Subjects follow the recommendations in treatments with a coordination mechanism that induces *unequal* expected payoffs (*CD90*) less frequently than in treatments with a coordination mechanism that induces *equal* expected payoffs (*CD50*).

More specifically, we expect that disadvantaged players are more sensitive to procedural unfairness than advantaged players. Following Bolton et al. (2005) and Trautmann (2009), we assume that individuals dislike being put at a disadvantage more than being in an advantaged position.

### **Hypothesis 4b**

Subjects in the role of the *disadvantaged* player follow the recommendations less frequently than the subjects in the role of the *advantaged* player in treatments with a coordination mechanism that induces unequal expected payoffs (*CD90*).

## Results

### Aggregate analysis

Table 4 presents mean values on contribution rates (playing *X*), coordination rates on one of the two pure-strategy NE (*X*, *Y*) or (*Y*, *X*), rates of following recommendations and point earnings across all subjects and rounds for each treatment. We use Wilcoxon signed-rank tests for testing single or matched samples and Wilcoxon rank-sum tests for testing unmatched samples using a 5% significance level, unless otherwise stated.^9^ Contribution rates amount to about 60% in all treatments, with no significant differences between treatments. However, due to the fact that subjects receive recommendations and follow them in more than 75% of cases, coordination rates are higher in treatments *CD50* and *CD90* compared to *Baseline*. The differences in coordination rates have an impact on efficiency in terms of earnings, which are lowest in *Baseline*, followed by *CD90*, and are highest in *CD50*.

As a robustness check, coordination rates and point earnings were also calculated using average values of the variables for all possible pairings, i.e. in each round we calculated for each subject in how many cases, out of possible 12 pairings, they would coordinate on one of two NE and what would be the corresponding payoff. The rates presented in the table are averages over these values over all 30 rounds. The results are similar to the values calculated based on the realized pairings, with one exception concerning the difference in earnings. Based on all possible pairings, earnings in *CD90* are not significantly higher than earnings in *Baseline* but lower than in *CD50*, although this difference is only weakly significant. This change in significance motivates a more detailed discussion of individual and total earnings in Sect. 5.2, where regression results confirm the results from realized pairings.

**Table 4:** Key variables in all treatments and pairwise comparisons

	Means			*p*-values of pairwise comparisons		
	*Baseline*	*CD50*	*CD90*	*Baseline*-*CD50*	*Baseline*-*CD90*	*CD50*–*CD90*
Contribution rate	0.614	0.577	0.580	0.172	0.754	0.302
Coordination rate	0.447	0.657	0.619	<0.001	<0.001	0.553
Coordination rate*	0.471	0.657	0.617	<0.001	0.002	0.783
Following rate	–	0.787	0.754	–	–	0.533
Earnings	5.308	6.171	5.991	<0.001	0.005	0.134
Earnings*	5.428	6.167	5.981	<0.001	0.355	0.069

Pairwise comparisons use Wilcoxon rank-sum tests. For tests on following rates and and earnings, data was collapsed at the subject level ($n=72$ in each treatment). Since coordination rates are the same for both types of players, $n=36$ in each treatment

* Values based on all possible pairings

As subjects were randomly matched in every round and no feedback was supplied, we do not expect any learning over time. Figures illustrating the averages of coordination rates and the decisions to follow the recommendations over the course of 30 rounds in different treatments can be found in the electronic supplementary material. Using Mann-Kendall tests, we do not find evidence for monotonic time trends, which allows us to aggregate the round-level data at the subject level for analysis.

**Fig. 1:**
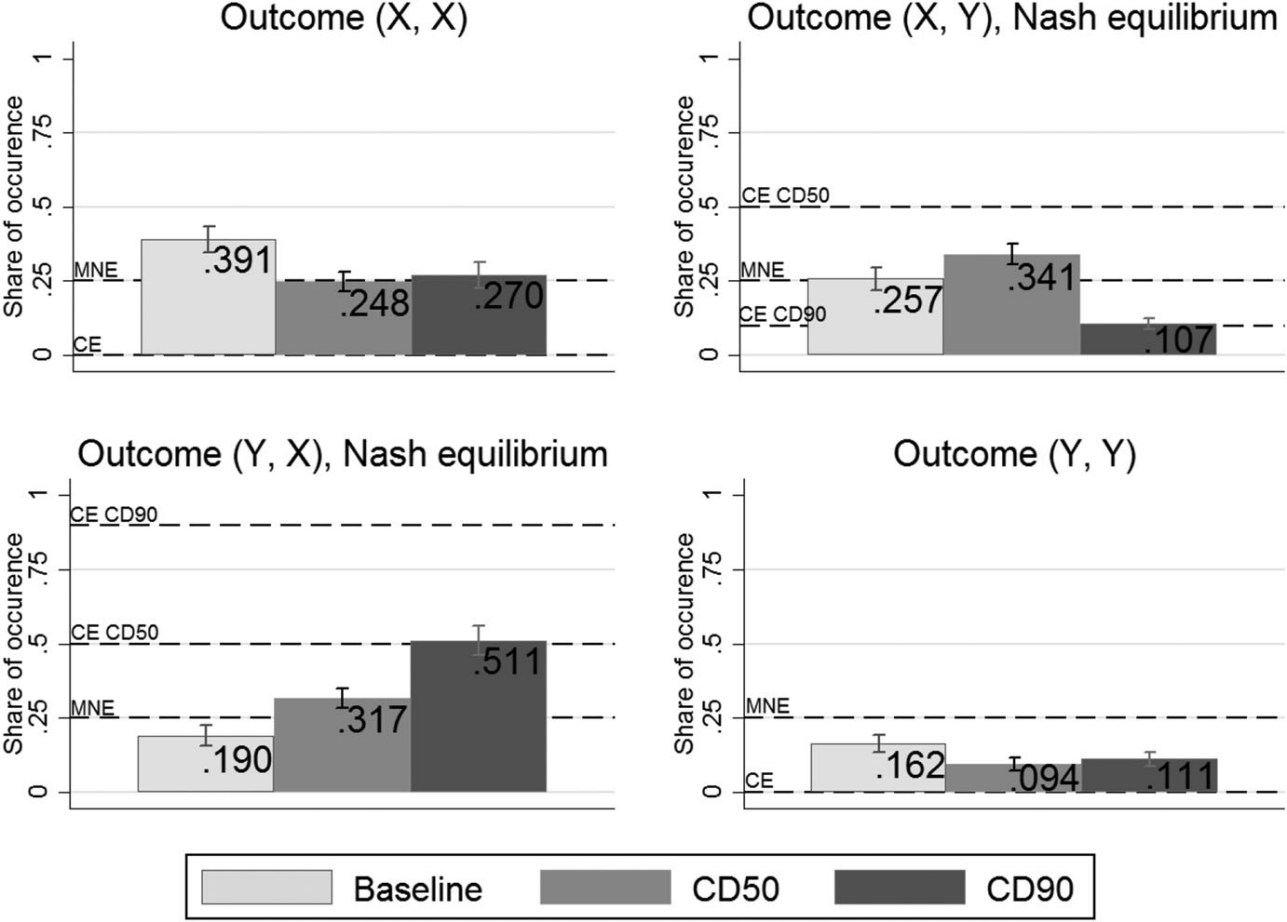
Outcomes played across treatments with MNE and CE predictions and 95% confidence intervals

Figure 1 gives a more detailed overview of play across treatments, comparing the observed frequencies of the four possible outcomes with the predictions of MNE and CE in the treatments *CD50* and *CD90*. Players in *Baseline* coordinate on one of the efficient outcomes significantly less often than 0.5, the rate predicted by MNE (${p}=0.016$).^10^ Both in *CD50* and *CD90*, coordination rates are significantly higher than in *Baseline* and higher than 0.5: 0.657 and 0.619 respectively (${p}<0.001$ for each comparison). Surprisingly, coordination rates between the treatments with recommendations are not significantly different from each other (see Table 4).

Figure 1 shows that players did not follow the recommendations all the time (${p}<0.001$ for both treatments), as the predicted outcome frequencies of the two NE were not reached. An exception is outcome (*X*, *Y*) in *CD90*, which was expected to be reached 10% of the time. Giving recommendations substantially reduces both under-provision (*Y*, *Y*) and over-provision (*X*, *X*) compared to *Baseline*, although levels of over-provision are still relatively high. This can be explained by the fact that players chose strategy *X*, which guaranteed a low payoff, more frequently than the payoff-uncertain strategy *Y* that resulted in higher payoff if both players followed their recommendations, but in a payoff of zero if the recommendation *X* was not followed.^11^

**Fig. 2:**
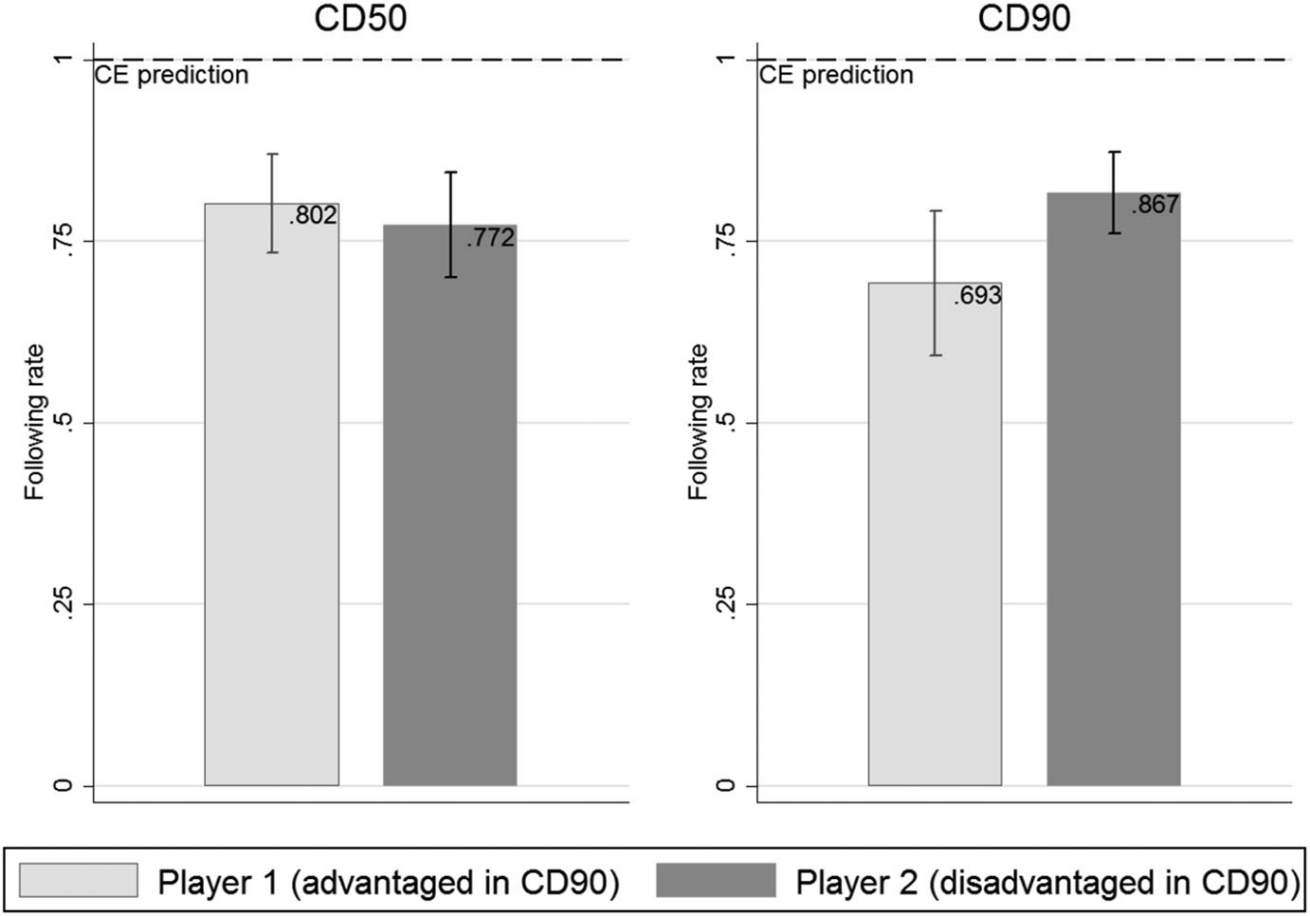
Following rates by player type in treatments *CD50* and *CD90* with 95% confidence intervals

Figure 2 illustrates to which extent the recommendations are followed by treatment and player type. We find no significant differences between treatments or player types. On average, 79% of all recommendations were followed in *CD50*, while 75% were followed in *CD90*.

Results of pairwise comparisons of average earnings between treatments are provided in the last rows of Table 4. As earnings depend on the subjects’ ability to coordinate, the results reflect the findings on coordination. We find significant differences in average earnings between *Baseline* and *CD50* and in average earnings between *Baseline* and *CD90*, but no significant differences in earnings between the treatments with recommendations. Average earnings in the treatments with recommendations are significantly lower than predicted by CE; while average predicted expected earnings in both *CD50* and *CD90* are 7.5, subjects earned only 6.17 (${p}<0.001$) in *CD50* and 5.99 in *CD90*. However, this was still significantly more than predicted by MNE in both treatments (5 points, ${p}<0.001$).

**Fig. 3:**
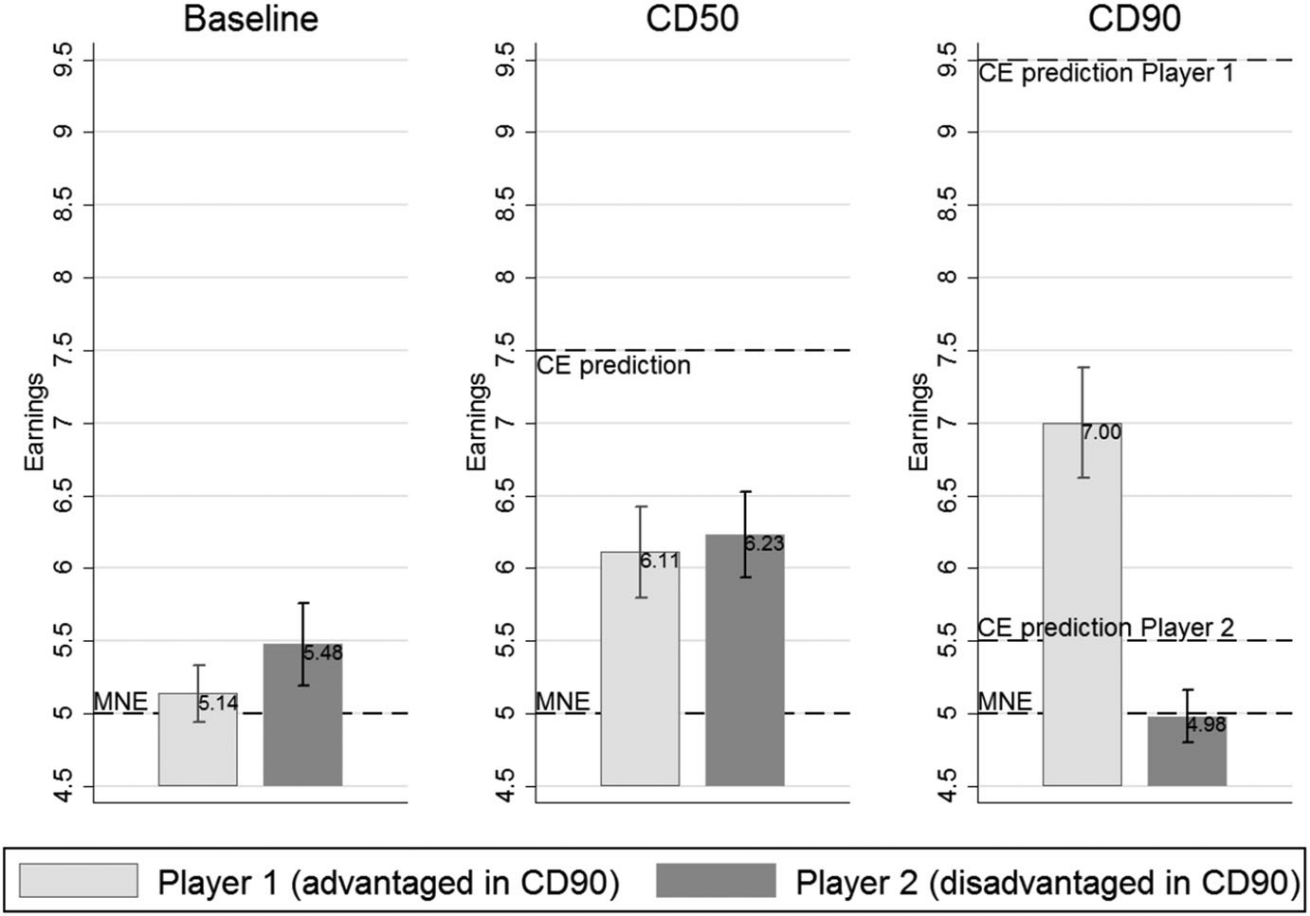
Earnings by player type in all treatments with 95% confidence intervals

Figure 3 presents mean earnings by player type and the comparison with predicted earnings of MNE and CE. In the baseline treatment, average earnings of type 1 players were 5.14 points, in line with MNE predictions, while they were significantly higher than the MNE prediction for type 2 players (5.48 points). However, the comparison of earnings between players shows no significant difference in payoffs between type 1 and type 2 players. In *CD50*, earnings of both players are not significantly different from each other, and lie between the earnings predicted by MNE and CE. In the treatment with unfair recommendations, type 2 players earned on average 4.98 points, which is close to what MNE predicts, but significantly lower than predicted by CE. Advantaged type 1 players earn 7.00 points, which is significantly different from MNE and CE predictions. From comparing these earnings with the theoretical benchmarks, we conclude that introducing an unfair procedure constitutes a Pareto improvement compared to a situation without any coordination procedure. Advantaged subjects were significantly better off, while disadvantaged subjects did not lose compared to MNE predictions.^12^

These findings lead us to the first three results: we do not reject Hypotheses 1 and 2a, but we reject Hypotheses 2b and 3:

#### **Result 1**

Action recommendations that induce equal expected payoffs for both players improve coordination rates and earnings compared to the situation without recommendations.

#### **Result 2**

Action recommendations that favour one of the players while putting the other one at a disadvantage improve coordination rates and earnings compared to the situation without recommendations.

#### **Result 3**

There are no significant differences in coordination rates and average earnings between treatments with fair and unfair recommendations. Hence, in aggregate terms, procedural fairness concerns seem to play a less important role than efficiency concerns.

### Analyses of individual following behaviour and individual earnings

Next, we examine individual determinants of the decision whether to follow a recommendation or not. Descriptive statistics on the subjects’ characteristics across treatments can be found in the electronic supplementary material. Randomization of subjects into treatments was successful, except for differences in the share of females and economics students. Thus, we will control for these variables in our regressions.

Table 5 shows the results of linear probability model (LPM) regressions with the dependent variable taking value 1 if a player followed the recommendation and 0 otherwise.^13^ In Model 1, individuals’ behaviour is explained by treatment and type of player, as well as the interaction between the two. Type 1 players, who were advantaged by the coordination mechanism in *CD90*, are less likely to follow the recommendation than type 1 players in *CD50*, although this effect is only marginally significant. There are no significant differences in following recommendations between type 2 players in *CD50* and *CD90* (${p}=0.321$). Testing the linear combination of parameters reveals that disadvantaged players in *CD90* follow the recommendations more often than advantaged players in *CD90* (${p}=0.028$).

Model 2 includes a dummy variable capturing if a subject received a favourable recommendation not to volunteer (i.e., to play *Y*) and its interaction with the treatment variable. This is the recommendation that potentially results in a payoff of 10, given both players follow their recommendation. However, if the other player does not follow the recommendation to volunteer, both players will earn zero points. Following a *Y*-recommendation thus always comes with the uncertainty of receiving zero. Players are significantly less likely to follow recommendation *Y* compared to recommendation *X*. While individuals are averse towards the possibility of getting zero payoff, procedural (un)fairness does not significantly affect one’s decision to follow a *Y*-recommendation, as the interaction effect between *CD90* and receiving an advantageous recommendation is very close to zero. Once the variable capturing the type of recommendation is included, the coefficient of the interaction term between treatment and player type becomes insignificant, indicating that the difference in the behaviour of type 1 players between treatments stems mainly from the fact that type 1 players in *CD90* receive more advantageous recommendations than type 1 players in *CD50*. There are no significant differences across players within or between the treatments with recommendations. One possible interpretation might be that it is not the unfair procedure per se that decreases the likelihood of following, but the uncertainty of the outcome. Individuals are willing to reject the favourable procedure to secure a lower payment, instead of dealing with the uncertainty if the other player will follow a recommendation that puts her at a disadvantage. Our results are in line with Van Huyck et al. (1990), who study how individuals behave when facing strategic uncertainty in coordination games with multiple equilibria and found support for individuals choosing actions that maximize minimum payoffs. In our study, strategy *X* is a maximin strategy, as volunteering ensures that the public good is provided and hence grants payoff of 5 to the provider.

To explore whether beliefs about others’ behaviour regarding recommendations affect the decision to follow recommendations across treatments and player types, we include a variable that captures subjects’ beliefs about how others react to recommendations (Model 3). This variable was elicited via the non-incentivized post-experimental questionnaire. In line with previous research (Cason and Sharma 2007), beliefs matter for individual behaviour. Those who believe that individuals in the role of the other player follow recommendations are more likely to follow them as well. It is also a sign that subjects understood that it is best for them to follow the recommendations if others do so.^14^

In the specification of Model 4, we control for the following variables: gender, period effects, session effects and subject of studies, as well as risk aversion and personality traits (measured by Locus of Control and Big Five tests), which seem not to be correlated with following the recommendations and have a very small effect on the coefficients of the other variables as well as on the goodness of fit.^15^ It might seem surprising that elicited risk preferences are not significant in explaining the decision to follow recommendations, which entails strategic uncertainty, but similar results have been found in previous studies. For example, Kocher et al. (2015) show that there is no relation between risk preferences and cooperation in a public good game. The authors argue that preferences towards risk stemming from nature might differ from the preferences towards uncertainty resulting from actions of another person (see also Bohnet et al. 2008).^16^

The analysis of individual-level behaviour in response to recommendations leads to results 4a and 4b, in which we reject hypotheses 4a and 4b:

**Table 5:** Linear probability model on following the recommendations

	Model 1 coef./SE	Model 2 coef./SE	Model 3 coef./SE	Model 4 coef./SE
Treatment				
CD90	−0.109*	−0.036	0.028	−0.001
	(0.059)	(0.058)	(0.056)	(0.073)
Type of player				
Player 2	−0.030	−0.030	−0.006	−0.037
	(0.048)	(0.048)	(0.040)	(0.040)
Treatment*Type of player				
CD90 × player 2	0.154**	0.020	−0.020	0.007
	(0.074)	(0.068)	(0.056)	(0.057)
Advantageous recomm.				
Yes		−0.154***	−0.154***	−0.154***
		(0.046)	(0.046)	(0.046)
Treatment*Advantageous recomm.				
CD90 × yes		−0.013	−0.013	−0.013
		(0.071)	(0.071)	(0.071)
Others follow				
Yes			0.288***	0.275***
			(0.034)	(0.035)
Constant	0.802***	0.879***	0.639***	0.764***
	(0.033)	(0.035)	(0.044)	(0.165)
Control variables	No	No	No	Yes
Adj. R^2^	0.012	0.036	0.128	0.142
Number of cases	4320	4320	4320	4320

Control variables include round, session dummies, female dummy, economics/business student dummy, below-average risk aversion dummy, Locus of Control, Big Five

Significance levels * 10%, ** 5%, *** 1%. Standard errors clustered at the subject level

#### **Result 4a**

In the treatment with the coordination mechanism that induces unequal expected payoffs (*CD90*), subjects do not follow the recommendations less than in the treatment with a coordination mechanism that induces equal expected payoffs (*CD50*).

#### **Result 4b**

Disadvantaged players do not follow recommendations significantly less often than advantaged players or players in the fair treatment. However, there are differences in how players react to advantageous recommendations: these are followed less often than disadvantageous recommendations.

We also conducted OLS regressions on individual point earnings. The results in Table 6 corroborate previous findings: following recommendations is a payoff-enhancing strategy for all players. Advantaged players in *CD90* earn significantly more than disadvantaged players in *CD90* and type 1 players in *CD50*, who in turn earn more than type 1 players in *Baseline*; type 2 players in *CD50* earn more than type 2 players in *Baseline* or disadvantaged players in *CD90*. There are no significant differences in payoffs between disadvantaged players in *CD90* and type 2 players in the *Baseline* treatment.

We further test the effects of the treatments on total earnings as a robustness check to our findings in Table 4. For Model 1 and Model 2 we create a hypothetical player whose earnings is the average of type 1 and type 2 players and calculate the marginal effects of the different treatments. For Model 1 the marginal effects are 0.181 for *CD50* compared to *CD90* (${p}=0.219$) and $-0.683$ for *Baseline* compared to *CD90* (${p}<0.001$). For Model 2 the marginal effects are 0.267 for *CD50* compared to *CD90* (${p}=0.345$) and $-0.562$ for *Baseline* compared to *CD90* (${p}=0.018$). Using this approach, we further confirm our findings reported in Table 4 concerning earnings: there is no difference in average earnings between treatments with recommendations, however earnings in both treatments: *CD50* and *CD90* are significantly higher than earnings in *Baseline*.^17^

**Table 6:** OLS regressions on earnings

	Model 1 coef./SE	Model 2 coef./SE	Model 3 (CD50&CD90) coef./SE	Model 4 (CD50&CD90) coef./SE	Model 5 (CD50&CD90) coef./SE
Treatment					
CD50	0.972***	0.995***			
	(0.18)	(0.29)			
CD90	1.861***	1.730***	0.889***	1.149***	1.146***
	(0.21)	(0.29)	(0.24)	(0.14)	(0.19)
Type of player					
Player 2	0.338**	0.310**	0.120	0.191	0.121
	(0.17)	(0.15)	(0.21)	(0.13)	(0.14)
Treatment*Type of player					
CD50 × player 2	−0.218	−0.331			
	(0.27)	(0.26)			
CD90 × player 2	−2.356***	−2.336***	−2.139***	−2.504***	−2.414***
	(0.26)	(0.25)	(0.29)	(0.18)	(0.19)
Follow recommendations					
Yes				2.378***	2.344***
				(0.13)	(0.14)
Constant	5.139***	5.186***	6.111***	4.204***	4.187***
	(0.10)	(0.57)	(0.15)	(0.14)	(0.46)
Control variables	No	Yes	No	No	Yes
Adj. R^2^	0.050	0.059	0.055	0.160	0.161
Number of cases	6480	6480	4320	4320	4320

Control variables include round, session dummies, gender, economics/business student dummy, below-average risk aversion dummy, Locus of Control, Big Five

Significance levels * 10%, ** 5%, *** 1%. Standard errors clustered at the subject level

### The role of beliefs

Our analysis shows that beliefs play an important role for individual behaviour. We are now going to analyse the relationship between beliefs and the fairness of the recommendation procedure. Our dependent variable describing beliefs takes value 1 if a subject believes that everyone or most of the players in the other role follow recommendations. There is a significant relationship between treatment and beliefs (chi-square test ${p}=0.042$), 64% of players in *CD90* believe that players in the other role will follow the recommendations, while it is 79% of all players in *CD50*. A further decomposition of data by type of player shows that these differences in beliefs are driven by type 1 players. 75% believe that others follow in *CD50*, while it is only 61% in *CD90* (${p}=0.035$ for the sub-sample of type 1 players). Hence, the advantaged players, knowing that others are put at a disadvantage, expect them to follow the recommendations less frequently. This may indicate that those players believe that disadvantaged players are concerned about the fairness of the procedure.

Beliefs of players correspond well with observed behaviour, with an exception for advantaged players in *CD90*: disadvantaged players in *CD90* follow recommendations significantly more often than advantaged players believe them to do (one-sided test of proportions ${p}=0.027$).

To investigate whether these differences in beliefs are related to individuals’ behaviour, we compare whether following rates differ with beliefs. Following rates are positively correlated with beliefs (${p}<0.001$ for each treatment). Players who believe that others follow, do follow themselves to a larger extent in both treatments (see Table 7). Conditional on individual beliefs, there are no differences in average following rates of both player types between treatments. The left panel of Table 7 displays following rates in treatment *CD50*, contingent on type of player and beliefs. In this treatment, both players were treated fairly by the coordination mechanism and their expected payoffs were the same; hence, we do not expect any differences in following the recommendation between players. Although there seems to be a small difference conditional on believing that others do not follow the recommendation, this is not statistically significant. The right panel of Table 7 shows following rates in treatment *CD90* contingent on beliefs and type of player. There is a significant difference in following recommendations between advantaged and disadvantaged players who think that other subjects mainly do not follow recommendations. Regardless of their beliefs, disadvantaged players follow their recommendations most of the time, while advantaged players only follow recommendations around 40% of the time if they believe others mostly do not follow.

**Table 7:** Following rates contingent on subjects’ beliefs and player type in *CD50* and *CD90*

Others follow	CD50		Wilcoxon rank-sum *p*	CD90		Wilcoxon rank-sum *p*
	Type of player			Type of player		
	Player 1	Player 2		Player 1 (advantaged)	Player 2 (disadvantaged)	
Yes	0.841	0.851	0.636	0.873	0.854	0.495
	$\hbox {n}=30$	$\hbox {n}=27$		$\hbox {n}=22$	$\hbox {n}=24$	
No	0.606	0.537	0.120*	0.410	0.742	0.001
	$\hbox {n}=6$	$\hbox {n}=9$		$\hbox {n}=14$	$\hbox {n}=12$	
Wilcoxon rank-sum *p*	0.008	<0.001		<0.001	0.050	

* *p*-value based on the exact statistic, since the number of observations in two groups is below 25

**Fig. 4:**
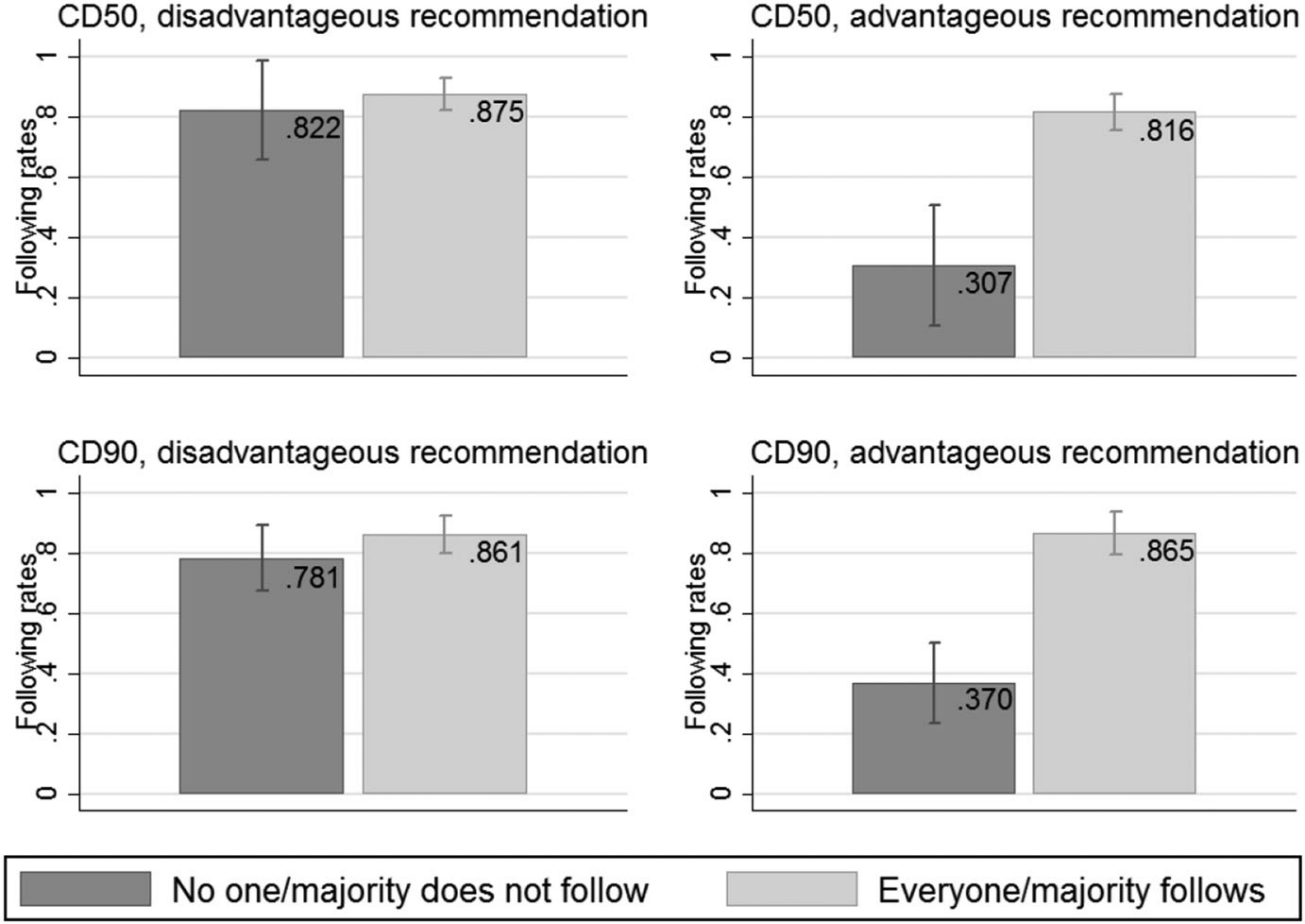
Following rates contingent on subjects’ beliefs and type of recommendation in *CD50* and *CD90* with 95% confidence intervals

Next, we look at following rates as a response to either a disadvantageous or advantageous recommendation. Figure 4 provides following rates for different types of recommendations contingent on beliefs. Recommendations to volunteer (*X*) are followed around 80% of the time, regardless of beliefs (see the left panel). If a player receiving recommendation *X* believes that her counterpart does not follow the received recommendation, not following her own recommendation involves the risk of getting zero, and apparently this risk outweighs the chance of getting the higher payoff. Beliefs are correlated with the decision to follow only when individuals receive the advantageous recommendation *Y* that involves the risk of getting zero payoff, as can be seen from the right panel. Players follow that recommendation significantly more often if they believe players in the other role do follow their recommendation as well.

These findings lead us to the following result:

#### **Result 5**

Advantaged individuals in the treatment with an unfair coordination mechanism believe less frequently that everyone or most of their counterparts follow recommendations than individuals in the treatment with a fair coordination mechanism. Furthermore, beliefs are correlated with following rates only when following the recommendation does not guarantee a safe payoff.

## Discussion and conclusion

Our study highlights the benefits of external action recommendations in improving coordination. We demonstrate that the existence of such a coordination mechanism increases efficiency, even if one party is strongly favoured by the mechanism. When individuals are confronted with a situation in which they face uncertainty about the behaviour of the other party, recommendations play an important role for coordination, even if it induces inequality in expected payoffs.

The findings from the study can be applied in coordination mechanisms where fairness might play a role, for example, informal rules governing the exploitation of common pool resources. While there might be many outcome allocations that guarantee sustainability, inequality in the expected harvest can lead to destabilization of the governing institutions (Klain et al. 2014; Cox et al. 2010). On a larger scale, preventing the catastrophic consequences of climate change can be modelled as a coordination game with multiple equilibria (Tavoni et al. 2011; DeCanio and Fremstad 2013; Madani 2013). In this context, action recommendations can be understood as the suggestion of an equilibrium profile by a ‘global planner’ (Forgó et al. 2005). This suggestion does not necessarily have to imply equal expected payoffs (Beg et al. 2002; Thomas and Twyman 2005). A negotiation process that is perceived as fair by all parties has been identified as an important prerequisite to reach an agreement (Winkler and Beaumont 2010; Lange et al. 2010; Rübbelke 2011).

We find that subjects follow disadvantageous recommendations more frequently than advantageous ones, which is in line with the results of Eckel and Wilson (2007), who show that signals of actions that are less risky but lead to a Pareto-inferior NE are more likely to be followed in a coordination game, compared to signals aiming at implementing a Pareto-superior NE involving more payoff-uncertainty. The authors find that signals to play the less risky but inefficient action are readily followed. Similarly, Brandts and Macleod (1995) find that the choice of strategy is affected by the minimum payoff that one can gain by playing it in a coordination game with recommended play. In other words, less risky strategies involving less payoff-uncertainty are more likely to be followed even if they constitute Pareto-inferior equilibria.

Our results corroborate findings of Hong et al. (2015). In their experiment, subjects had to trade off a fair distribution of payoffs against an increasing sum of payoffs. The authors estimate social welfare preferences and find that the majority of the individuals weakly prefers efficiency over equality.

However, our findings differ from Anbarci et al. (2017) where subjects received external recommendations that implied ex-ante payoff-equality but ex-post inequality in Battle of the Sexes games. The authors report generally higher following rates than we do, but find that subjects disregard the recommendations more often when payoff-asymmetry increases. Potential reasons for these discrepancies can be found in differences in the experimental design. Firstly, Anbarci et al. use a game with two outcomes that imply zero payoff to both players. This might explain why they find higher following rates. A second difference is that subjects in their experiments receive feedback between interactions, which makes it possible for subjects to condition their following behaviour on past outcomes, which can make payoff differences more salient. Thirdly, they change the payoff matrix across treatments and keep the probabilities of their recommendations constant, while we keep the matrix constant and measure the impact of the fairness of the recommendation procedure. Our interpretation of the differing results of both studies is that individuals are more sensitive towards payoff (distributional) inequality than towards process inequality. Yet, for conclusive evidence further research has to be conducted explicitly comparing preferences for distributional fairness with preferences for procedural fairness.

Furthermore, the study by Bolton et al. (2005) can help explain the high acceptance of our unfair recommendation procedure. The authors show that a biased procedure is more likely to be accepted if an unbiased procedure is not feasible. In our study, subjects can either follow recommendations that put one of them in a disadvantaged position or reject it; however, rejection implies a substantial loss of efficiency. There is no fair coordination procedure available in treatment *CD90*. Potentially, if an unfair procedure was publicly chosen over the fair one, rejection rates of the recommendations could be higher.

Moreover, it is possible that subjects would reject unfair action recommendations to a larger extent if they were picked by other subjects instead of the experimenters, in a similar fashion as they reject unfair ultimatum proposals more often if they are chosen by a subject using a ‘monocratic’ rule compared to a ‘democratic’ rule, as for example in Grimalda et al. (2008). In our experiment, the procedure was chosen by the experimenter and subjects were randomized into the roles of player 1 and player 2. Randomization into roles could be seen as a fair procedure, reducing potential concerns about the lottery determining expected earnings. However, Bolton et al. (2005) observe rejections of unfair procedures even if they are implemented by an experimental lottery similar to our study. Furthermore, in a post-experimental questionnaire, we asked subjects if they feel disadvantaged and learned that significantly more type 2 players in *CD90* feel disadvantaged than type 1 players in the same treatment (Wilcoxon rank-sum test, ${p}<0.001$). More research is needed to identify the characteristics of situations in which unfair procedures are rejected versus situations in which such procedures are accepted. This is also crucial for policy-makers to understand when policy suggestions, for example on public good provision, will face resistance and when they will be accepted by the general public.

Our study is limited to cases that can be represented as one-shot situations, as subjects had only a low probability of encountering their current “partner” repeatedly in our experiments. It would be of interest to investigate in future research how outcomes change when subjects learn the outcomes after every encounter. It might well be the case that procedural fairness considerations become more salient when individuals are allowed to learn over time.

Our choice of game was guided by the non-existence of strictly dominated strategies, high potential gains from following the recommendations, and the applicability to threshold public good provision. However, we think that the influence of procedural fairness concerns can be important in other games as well. Examining the sensitivity of our results with respect to different types of games and payoff structure (e.g. by varying the difference between payoffs in case of coordination and miscoordination) is left to further research.

In general, the role of beliefs that are formed when individuals face different procedures deserves further investigation. In our study, beliefs were elicited only after the whole experiment in a non-incentivized task and were not contingent on the type of recommendation. Our results indicate that subjects might hold wrong beliefs about how others react to recommendations when facing a procedure treating individuals unequal. As incorrect beliefs can lead to further inefficiencies if subjects act in accordance with them, additional research is necessary to explore their role in driving people’s behaviour in situations in which concerns about procedural fairness and efficiency, as well as strategic uncertainty, are involved.

## Electronic supplementary material

Below is the link to the electronic supplementary material.

Supplementary material 1 (pdf 652 KB)

## Footnotes

1 For more theoretical and experimental results on the Volunteer’s Dilemma, see Darley and Latane (1968), Latane and Rodin (1969), Diekmann (1993), Weesie (1993, 1994) and Myatt and Wallace (2008).

2 For an overview on coordination failures in laboratory experiments, see Camerer (2003) and Devetag and Ortmann (2007).

3 Another way to manipulate procedural fairness in experiments is to vary the probability of the random draw that assigns the roles of the subjects in the experiment. Grimalda et al. (2016) find that individuals respond to the probability of being either the advantaged proposer or the disadvantaged receiver in a following Ultimatum game.

4 Our study hence uses a definition of fairness different from that of, for example, Kahneman et al. (1986), who define fairness in terms of reference points and framing, or, Konow (2001), who finds context-dependence of fairness. We limit our study to investigating procedural fairness as defined in the articles and models cited above.

5 In the experiment, names of actions were framed in a neutral way (*X*, *Y*) in order to avoid framing effects.

6 Before running the experiments, we conducted two pilot sessions of *CD50* and *CD90*. Subjects in these pilots had problems understanding the part of the instructions dedicated to the recommendations. As this part is central, we clarified it and supplied more information; for example, we explicitly stated that the probability that both matched participants at the same time get recommendations *X* or *Y* is zero.

7 The calibration used in the risk task has been introduced by Charness and Gneezy (2010).

8 The belief elicitation was conducted after the experiment and was not incentivized. The scale of four items with verbal descriptions of the others’ following behaviour was chosen for the belief elicitation to avoid potential problems with correct expression of probabilities among subjects; see Erev et al. (1993).

9 In all aggregate tests, we have pooled the data at the subject level across rounds to achieve independent observations. Furthermore, since coordination rates are the same for both types of players (a successful coordination by definition requires two players of different type matched with each other), we have run the tests only on the data from one type of player.

10 In *Baseline*, we cannot reject the hypothesis that outcome (*X*, *Y*) was achieved 25% of time, but we can reject this hypothesis for outcome (*Y*, *X*). This result is surprising, since outcomes (*X*, *Y*) and (*Y*, *X*) are perfectly symmetric. Even though it seems that type 1 players chose strategy *X* more frequently than type 2 players, we cannot reject the hypothesis that both types of players played *X* with equal proportions. One possible explanation is a relatively low number of observations; another possible reason might be the emergence of conventions that can differ between populations and is facilitated by labels (Van Huyck et al. 1997).

11 The hypothesis of equal frequencies of choosing *X* and *Y* is supported only for type 2 players in treatment *CD50*.

12 These results are robust to potential effects of the round matching by recalculating all possible earnings of players in *CD90*, as described in the introduction of this section.

13 Since we are mainly interested in interaction effects between treatment and type of player, we use LPM regressions to analyse the data, as resulting interaction terms cannot be interpreted in the same way in non-linear models as in linear models (for contributions to this discussion see e.g. Ai and Norton 2003; Greene 2010; Puhani 2012; Karaca-Mandic et al. 2012).

14 A more detailed analysis of the relevance of beliefs is conducted in the next subsection.

15 Only individuals with a more pronounced trait Neuroticism follow recommendations significantly less often (${p}=0.012$).

16 As robustness checks, we ran panel regressions, probit regressions and logistic regressions with odds ratios. Results are consistent with our LPM results and are available upon request. We also analysed whether there are significant gender differences between treatments. While women follow recommendations significantly more frequently than men in treatment *CD50*, the relation is insignificant in *CD90* and has the opposite sign. Furthermore there are no differences between women in *CD50* and *CD90*, nor between men in these conditions. To conclude, while women do follow recommendations more frequently than men if the procedure is fair, these gender differences disappear in an unfair environment. The results are available from the authors upon request.

17 We also estimated the marginal effects of treatments in OLS regressions based on all possible pairings of type 1 and type 2 players within a session. This approach gives us qualitatively identical results.
