# Supplementary material for: Fairness versus efficiency: how procedural fairness concerns affect coordination
Source: Exp Econ. 2017 Sep 4;21(3):601–26. doi: 10.1007/s10683-017-9540-5 (PMC6061030; doi:10.1007/s10683-017-9540-5)
Supplement: Supplementary file 1 — Supplementary material 1 (pdf 652 KB) [file 10683_2017_9540_MOESM1_ESM.pdf]

Electronic Supplementary Material to "Fairness versus efficiency: how procedural fairness concerns affect coordination" by Verena Kurz, Andreas Orland and Kinga Posadzy

Tables

Table 1: Balancing test on observables

	Means			Differences		
	Baseline	CD50	CD90	CD50-Baseline	CD90-Baseline	CD50-CD90
female	0.514	0.653	0.569	-0.139*	-0.055	0.083
age	24.46	23.96	23.54	0.500	0.917	0.417
semesters	4.028	4.403	4.028	-0.375	0	0.375
econ	0.472	0.278	0.431	0.194**	0.042	-0.153*
Risk aversion task	6.403	6.625	6.944	-0.222	-0.542	-0.319
Personality traits						
Locus of Control[a]	11.72	12.17	12.64	-0.444	-0.917	-0.472
Neuroticism[b]	12.17	13.03	12.31	-0.861	-0.139	0.722
Extraversion[b]	14.79	15.04	15.21	-0.250	-0.417	-0.167
Openness[b]	15.04	15.85	15.49	-0.806	-0.444	0.361
Agreeableness[b]	15.29	16.03	15.29	-0.736	0	0.736
Conscientiousness[b]	15.43	15.15	15.60	0.278	-0.167	-0.444
N	72	72	72			

Significance levels : $*$: 10% $**$: 5%. Two-sided t-tests.

[a] Locus of Control can range from 0 to 23. We added up the external answers to the questions, hence a higher LoC means that a subject is more external and believes that her life and decisions are controlled by environmental factors rather than by herself.

[b] Each of the Big Five traits can range between 3 and 21. We added up the answers given on seven-item Likert scales to the three questions for each trait. A higher score means that the trait is more pronounced.

Figures

Figure 1: The series of recommendations in *CD50* and *CD90* for Player 1

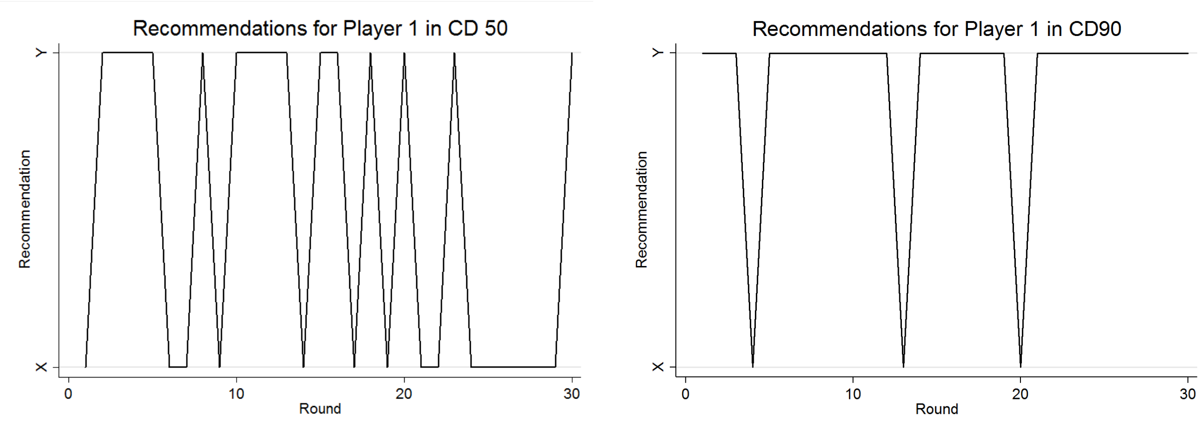

Figure 2: Average coordination rates over time in all three treatments

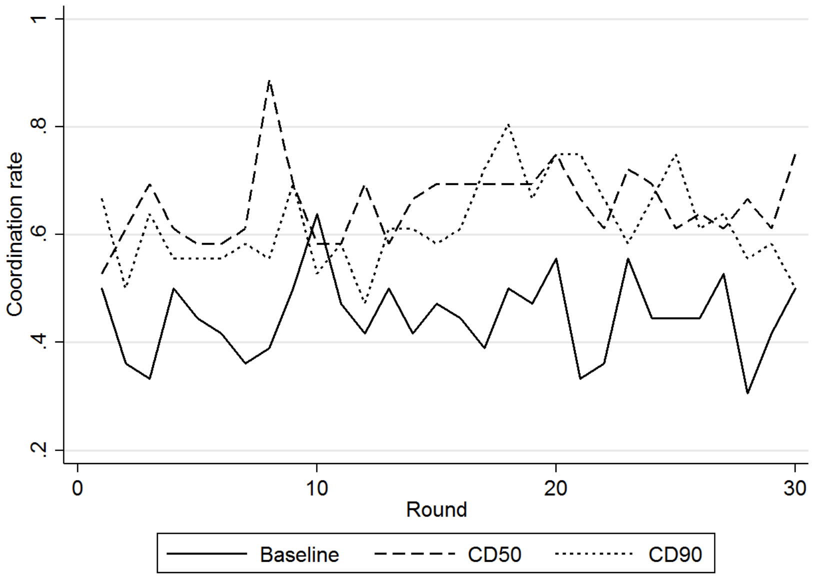

Figure 3: Average following rates over time in *CD50* and *CD90*

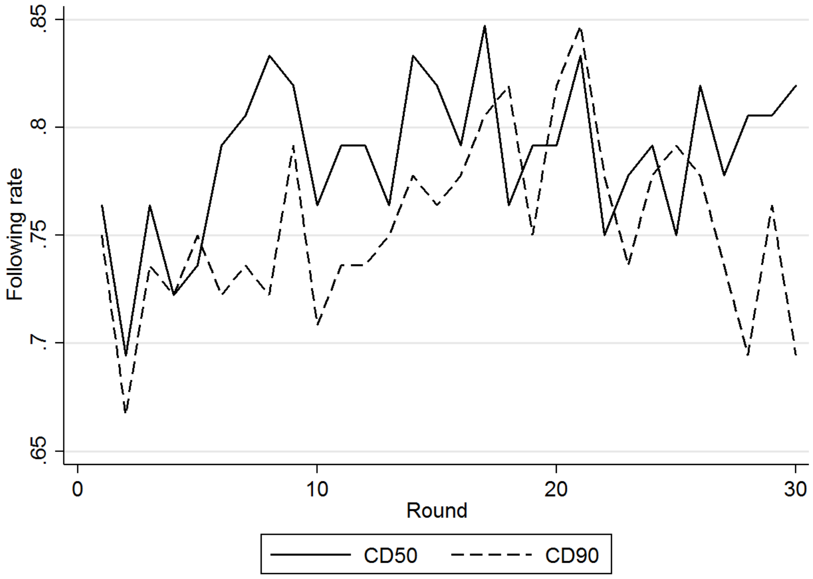

Figure 4: Average earnings over time in all three treatments

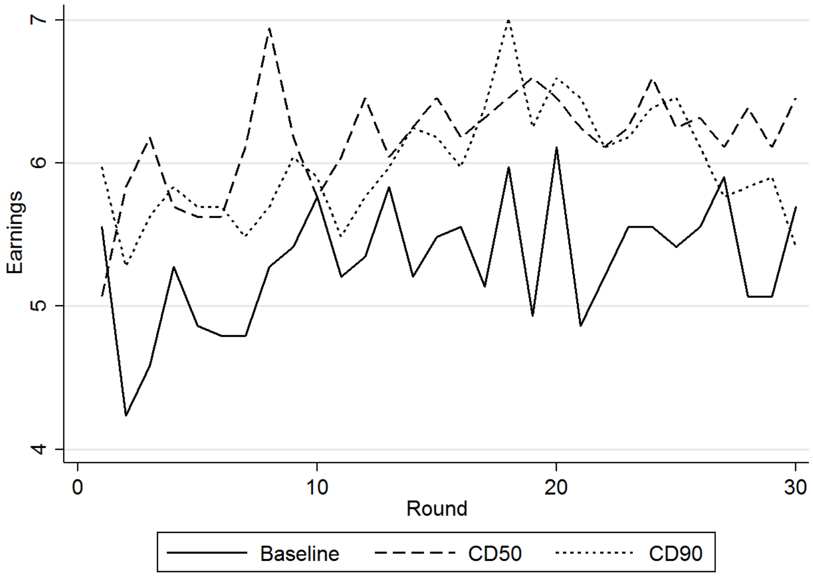

Figure 5: Average following rates over time in *CD50*, separately for Player 1 and Player 2

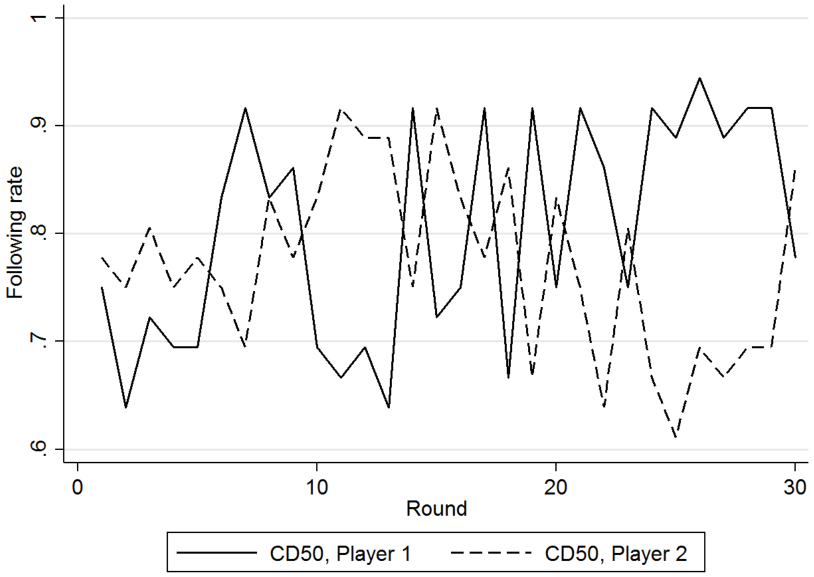

Figure 6: Average following rates over time in *CD90*, separately for Player 1 and Player 2

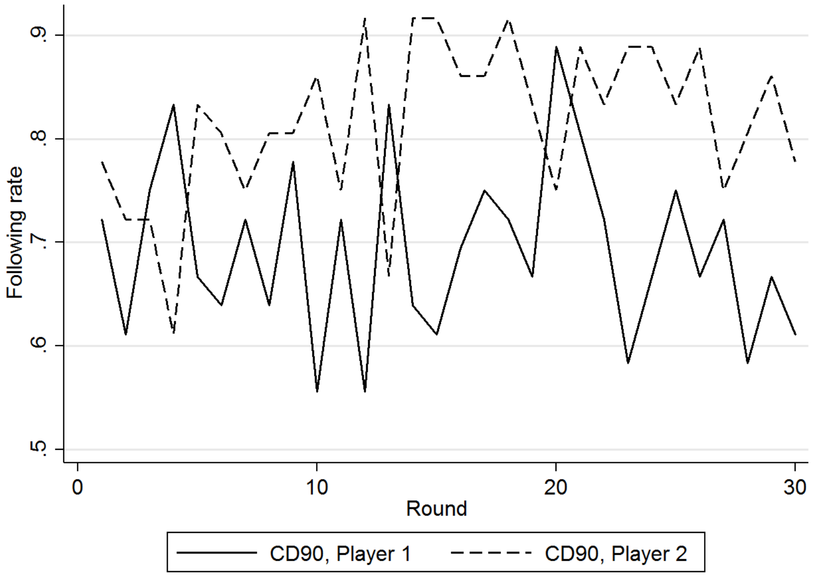

Translation of the Experimental Instructions for the *CD50* Treatment

General Instructions

Welcome to this experiment! Please read the instructions carefully. They are identical for all participants. During the experiment, you and the other participants are asked to make decisions. All money you earn will be paid to you privately in cash at the end of the experiment. In addition, you will receive a show-up fee of 4 euros.

During the experiment, it is forbidden to talk with the other participants, to use mobile phones, or to start other programs at the computer. Please also turn off all electronic devices. If you do not follow these rules, you will be excluded from the experiment and all payments.

If you have a question, please raise your hand. An experimenter will then come and answer your question quietly. If the question is relevant for all participants, we will repeat it publicly and answer it.

Part I of the experiment

Roles and the number of rounds

In this part of the experiment, you will be asked to make a decision in each of 30 rounds, which will be described below. 24 people participate in today's experiment. Before the first round begins, all participants will be randomly divided for today's experiment into two equal-sized groups. One group is called the Red Participants, and the other is called the Blue Participants. The group you are in will stay the same throughout the experiment.

In each round, you will be randomly matched to a person in the other group. You have an equal chance of 1-to-12 of being matched to any particular person in the other group. You will never interact with participants belonging to the same group as you. You will not be told the identity of the person you are matched with, nor will that person be told your identity, even after the end of the session. All the decisions you make, and the other information you provide us, will remain confidential.

The structure of the experiment in each round

All rounds are identically structured. Both you and the person you are matched with have two choices available: X and Y. The choices that you and your matched participant make jointly determine your point earnings for the round. The following table shows how the amount of points depending on your and your matched participant's decisions is determined:

Payment table

		**Blue Participant**	
		X	Y
**Red Participant**	X	Red earns: 5<br>Blue earns: 5	Red earnst: 5<br>Blue earnst: 10
	Y	Red earns: 10<br>Blue earns: 5	Red earns: 0<br>Blue earns: 0

In each round, one of the four cells in the above table will be relevant to your point earnings. If you are a Red Participant, your choice of X or Y will determine which row of the table

the relevant cell belongs to. Your matched participant's choice of X or Y will determines the column.
If you are a Blue Participant, the situation is reversed: your choice of X or Y will determine which column of the table the relevant cell belongs to, and your matched Red Participant's choice of X or Y will determine which row the relevant cell belongs to.
In both cases, your choices, as well as the choices of the participant you are matched with, determine the relevant cell. The first number in the relevant cell represents the Red Participant's point earning for the round and the second number represents the Blue Participant's point earning for the round.

- If the Red Participant chooses X and the blue participant chooses Y, Red earns 5 points and Blue earns 10 points.
- If both participants choose X, both each receive 5 points.
- If both participants choose Y, both each receive 0 points.
- If the Red Participant chooses Y and the blue participant chooses X, Red earns 10 points and Blue earns 5 points.

Recommendations

Before you choose your action for each round, both you and the participant you are matched with will be given recommendations on the screen. In any round, there are two possible recommendations. Those are generated according to the following rules:

- There is a 50% chance (on average 5 out of 10 times) in each round that it will be recommended that the Red Participant choose $\boldsymbol{X}$ and the Blue Participant choose $\boldsymbol{Y}$.
- There is a 50% chance (on average 5 out of 10 times) in each round that it will be recommended that the Red Participant choose $\boldsymbol{Y}$ and the Blue Participant choose $\boldsymbol{X}$.

It will never happen that you are recommended to both choose X or both choose Y . These recommendations are optional; it is up to you whether or not to follow them. Notice that your recommendation also gives you information about the recommendation that was given to the person matched to you. The recommendations themselves have no direct effect on the points you can earn. The following table summarizes these recommendations and their likelihoods.

		**Blue Participant**	
		X	Y
**Red Participant**	X	never recommended<br>Red earns=5,<br>Blue earns=5	recommended with 50% probability (5 out of 10 times)<br>Red earns=5,<br>Blue earns=10
	Y	recommended with 50% probability (5 out of 10 times)<br>Red earns=10,<br>Blue earns=5	never recommended<br>Red earns=0,<br>Blue earns=0

Your decision

Each participant makes his or her decision without knowing the decision of the other participant. The following figure shows the example of a screen-shot where you enter your decision:

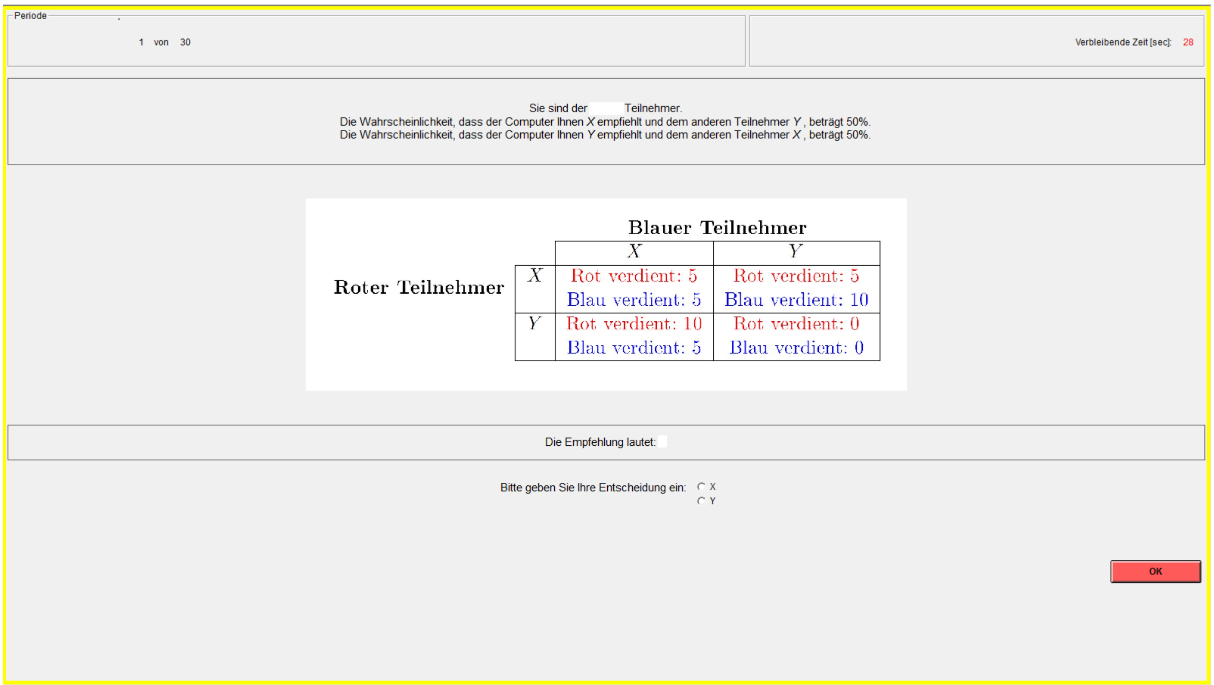

You should make your decision within the proposed 30 seconds. The computer program gives you as much time as you need, even though this takes more than the 30 seconds. After that time, you will be shown the request “Please make your decision now”.

After all participants have made their decisions and clicked the red OK button, the next round will start immediately. You will not receive any information on the decision of the other participant or the point earnings. This information will be provided to you after the end of the experiment.

As a reminder: You will be re-matched with a participant in the other role before each round.

Earnings from part I of the experiment

After round 30, the computer program will randomly select two rounds. The total number of points you earn in these two rounds will be converted into cash at an exchange rate of 75 euro cents per point. The two rounds chosen for the payments hold for all the participants. You will be informed at the end of the experiment which two rounds were chosen for payment.

Part II of the experiment

The second part of the experiment is independent from the first part. Both the instructions and the exchange rate from points to euros for part II will be different from part I. All necessary information and the exchange rate will be shown on the computer screen after the end of the first part. If you have questions concerning part II, raise your hand. An experimenter will then come to your place to answer your questions quietly.

After part II of the experiment

After the second part of the experiment, the computer will show a questionnaire. After you have filled in the questionnaire completely, you will see a summary of all your decisions and the decisions of the participants you were matched with. It will also show you your earnings. The earnings are calculated from the points you received in both parts of the experiment and the respective exchange rates. Your cash payment is this amount plus the 4 euros show-up fee.

If you have any questions, please raise your hand now. If there are no further questions, the experiment will start with a short quiz at the computer. This quiz is solely conducted to test your understanding of these instructions and has no influence on your payment.

Translation of the Questions with Correct Answers of the On-screen Quiz in the *Baseline* Treatment

- Right or wrong: I stay in all 30 rounds a Red or Blue participant. (Right)
- Right or wrong: I will meet in all 30 rounds the same participant in the other role. (Wrong)
- Right or wrong: I can observe the other participant's choice of X or Y before I make my own choice of X or Y. (Wrong)
- Assume you are the Red participant. If you choose X and the other participant Y, what are your point earnings? (5)
- Assume you are the Blue participant. If you choose Y and the other participant X, what are your point earnings? (10)
- Assume that you and the other participant choose X. How many points earns each of you? (5)
- Assume that you and the other participant choose Y. How many points earns each of you? (0)
- Right or wrong: At the end of the experiment I receive the earnings of two randomly chosen rounds in part I of the experiment at an exchange rate of 0.75 euros per point. (Right)

Translation of the Questions with Correct Answers of the On-screen Quiz in the *CD50* Treatment

- Right or wrong: I stay in all 30 rounds a Red or Blue participant. (Right)
- Right or wrong: I will meet in all 30 rounds the same participant in the other role. (Wrong)
- Right or wrong: If the recommendation of my computer is X, then the other participant's recommendation is also X. (Wrong)
- What is the probability of receiving a Y recommendation for Red participants? (50
- What is the probability of receiving an X recommendation for Red participants? (50
- Out of 10 recommendations, how many X recommendation sees a Red participant on average? (5)
- Out of 10 recommendations, how many Y recommendation sees a Red participant on average? (5)
- What is the probability of receiving a Y recommendation for Blue participants? (50
- What is the probability of receiving an X recommendation for Blue participants? (50
- Out of 10 recommendations, how many X recommendation sees a Blue participant on average? (5)
- Out of 10 recommendations, how many Y recommendation sees a Blue participant on average? (5)
- Right or wrong: I can observe the other participant's choice of X or Y before I make my own choice of X or Y. (Wrong)
- Assume you are the Red participant. If you choose X and the other participant Y, what are your point earnings? (5)
- Assume you are the Blue participant. If you choose Y and the other participant X, what are your point earnings? (10)
- Assume that you and the other participant choose X. How many points earns each of you? (5)
- Assume that you and the other participant choose Y. How many points earns each of you? (0)
- Right or wrong: At the end of the experiment I receive the earnings of two randomly chosen rounds in part I of the experiment at an exchange rate of 0.75 euros per point. (Right)

Translation of the Questions with Correct Answers of the On-screen Quiz in the *CD90* Treatment

- Right or wrong: I stay in all 30 rounds a Red or Blue participant. (Right)
- Right or wrong: I will meet in all 30 rounds the same participant in the other role. (Wrong)
- Right or wrong: If the recommendation of my computer is X, then the other participant's recommendation is also X. (Wrong)
- What is the probability of receiving a Y recommendation for Red participants? (90
- What is the probability of receiving an X recommendation for Red participants? (10
- Out of 10 recommendations, how many X recommendation sees a Red participant on average? (1)
- Out of 10 recommendations, how many Y recommendation sees a Red participant on average? (9)
- What is the probability of receiving a Y recommendation for Blue participants? (10
- What is the probability of receiving an X recommendation for Blue participants? (90
- Out of 10 recommendations, how many X recommendation sees a Blue participant on average? (9)
- Out of 10 recommendations, how many Y recommendation sees a Blue participant on average? (1)
- Right or wrong: I can observe the other participant's choice of X or Y before I make my own choice of X or Y. (Wrong)
- Assume you are the Red participant. If you choose X and the other participant Y, what are your point earnings? (5)
- Assume you are the Blue participant. If you choose Y and the other participant X, what are your point earnings? (10)
- Assume that you and the other participant choose X. How many points earns each of you? (5)
- Assume that you and the other participant choose Y. How many points earns each of you? (0)
- Right or wrong: At the end of the experiment I receive the earnings of two randomly chosen rounds in part I of the experiment at an exchange rate of 0.75 euros per point. (Right)
